# Characterization of brain tumor initiating cells isolated from an animal model of CNS primitive neuroectodermal tumors

**DOI:** 10.18632/oncotarget.24460

**Published:** 2018-02-09

**Authors:** Sergey Malchenko, Simone T. Sredni, Jerusha Boyineni, Yingtao Bi, Naira V. Margaryan, Maheedhara R. Guda, Yulia Kostenko, Tadanori Tomita, Ramana V. Davuluri, Kiran Velpula, Mary J.C. Hendrix, Marcelo B. Soares

**Affiliations:** ^1^ Department of Cancer Biology & Pharmacology, University of Illinois College of Medicine, Peoria, Illinois, United States of America; ^2^ Department of Surgery, Division of Pediatric Neurosurgery, Feinberg School of Medicine, Northwestern University, Chicago, Illinois, United States of America; ^3^ Cancer Biology and Epigenomics Program at The Stanley Manne Children’s Research Institute, Ann & Robert H. Lurie Children’s Hospital of Chicago, Chicago, Illinois, United States of America; ^4^ Department of Preventive Medicine, Division of Health and Biomedical Informatics, Feinberg School of Medicine, Northwestern University, Chicago, Illinois, United States of America; ^5^ AbbVie Bioresearch Center, Worcester, Massachusetts, United States of America; ^6^ Department of Biochemistry, Robert C. Byrd Health Sciences Center and Cancer Institute, West Virginia University, Morgantown, West Virginia, United States of America; ^7^ Department of Biology, Shepherd University, Shepherdstown, West Virginia, United States of America

**Keywords:** CNS-PNET animal model, radial glia, brain tumor-initiating cells, RNA/DNA-seq, gene signature

## Abstract

CNS Primitive Neuroectodermal tumors (CNS-PNETs) are members of the embryonal family of malignant childhood brain tumors, which remain refractory to current therapeutic treatments. Current paradigm of brain tumorigenesis implicates brain tumor-initiating cells (BTIC) in the onset of tumorigenesis and tumor maintenance. However, despite their significance, there is currently no comprehensive characterization of CNS-PNETs BTICs. Recently, we described an animal model of CNS-PNET generated by orthotopic transplantation of human Radial Glial (RG) cells - the progenitor cells for adult neural stem cells (NSC) - into NOD-SCID mice brain and proposed that BTICs may play a role in the maintenance of these tumors. Here we report the characterization of BTIC lines derived from this CNS-PNET animal model. BTIC’s orthotopic transplantation generated highly aggressive tumors also characterized as CNS-PNETs. The BTICs have the hallmarks of NSCs as they demonstrate self-renewing capacity and have the ability to differentiate into astrocytes and early migrating neurons. Moreover, the cells demonstrate aberrant accumulation of wild type tumor-suppressor protein p53, indicating its functional inactivation, highly up-regulated levels of onco-protein cMYC and the BTIC marker OCT3/4, along with metabolic switch to glycolysis - suggesting that these changes occurred in the early stages of tumorigenesis. Furthermore, based on RNA- and DNA-seq data, the BTICs did not acquire any transcriptome-changing genomic alterations indicating that the onset of tumorigenesis may be epigenetically driven. The study of these BTIC self-renewing cells in our model may enable uncovering the molecular alterations that are responsible for the onset and maintenance of the malignant PNET phenotype.

## INTRODUCTION

CNS Primitive Neuroectodermal tumors (CNS-PNETs) are members of the embryonal family of malignant childhood brain tumors, which remain refractory to current therapeutic treatments [1].

There is a paradigm of brain tumorigenesis that implicates a limited number of genomic and/or epigenomic alterations in the transformation of neural stem cells (NSC) into brain tumor-initiating cells (BTIC) [2–5]. In particular, Radial Glial (RG) cells - the progenitor cells for the adult NSCs - are considered as BTICs of ependymoma, a brain tumor of glial origin [2, 3]. In a recent study, it was shown that CNS-PNET BTICs derived from a clinical specimen were able to maintain neuronal and glial differentiation and demonstrated a self-renewal potential - the hallmarks of NSCs [6]. However, despite their role in tumor maintenance, there is no comprehensive characterization of CNS-PNETs BTICs to date.

Recently, we described an animal model of CNS-PNET that was generated by orthotopic transplantation of human Radial Glial (RG) cells - the progenitor cells for adult NSCs - into NOD-SCID mice sub-ventricular zone of the brain [7], and proposed that BTICs may play a role in the maintenance of these tumors [8]. We documented expression of RG-BTIC markers such as SOX2, Vimentin and Nestin, BTIC marker OCT3/4, up-regulation of onco-protein cMyc, along with an aberrant accumulation of stabilized tumor-suppressor protein p53 in the model tumors [8].

Here we report the characterization of BTICs derived from CNS-PNETs in our animal model. The main objectives of this study were to investigate whether genomic alterations are involved in the process of RG transformation into BTICs and to uncover differences in gene expression level of known cancer related genes between the RG and the correspondent BTICs, thus contributing to a better understanding of the key function of these cells in tumor maintenance.

## RESULTS

### BTIC derivation

**Figure 1:**
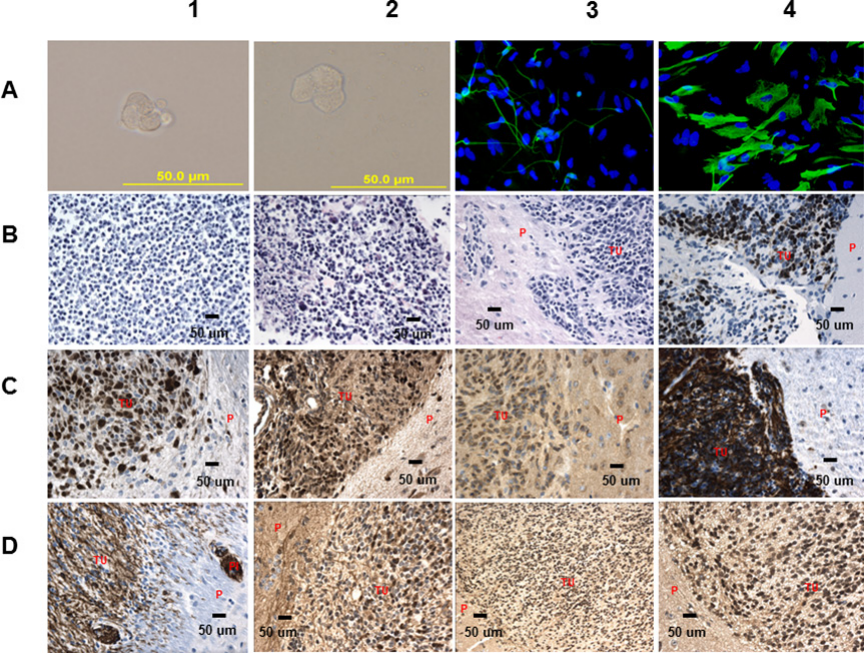
Analysis of RG, TCL and the TCL derived tumors **(A1, A2)** LCAS-R 2nd nsphrs/LCAS-RTL(138) 2nd nsphr, **(A3)** Tubb3 40x LCAS-RTL(138) 2nd nsphr, **(A4)** GFAP 40x LCAS-RTL(138) 2nd nsphr; **(B1, B2)** HE 40x LCAS-R/LCAS-RTL(138) - images show undifferentiated small round blue cells with scarce cytoplasm, **(B3)** HE 40x (LCAS-RTL(138) SVZ 15 weeks post-injection) - extensive proliferation of undifferentiated embryonal tumor cells infiltrating the adjacent brain parenchyma, **(B4)** Ki67 40x (LCAS-RTL(138) SVZ 15 weeks post-injection) - high proliferative activity of tumor cells is reflected by over 75% of cells expressing Ki-67, **(C1)** OCT3/4 40x (LC26-RTL(170) SVZ 12 weeks post-injection) - extensive proliferation of tumor cells invading adjacent brain parenchyma show high expression of OCT3/4 (over 80% of cells), **(C2)** Sox2 40x (LC26-RTL(170) SVZ 12 weeks post-injection) - extensively expressed in the tumor cells, **(C3)** PRAME 40x (LCAS-R 12 weeks post-injection) - extensively expressed in the tumor cells, **(C4)** Nestin 40x (LCAS-RTL(138) 15 weeks post-injection) - extensively expressed in the tumor cells, **(D1)** Vimentin 40x (LCAS-RTL(138) 15 weeks post-injection) - extensively expressed in the tumor cells within the main tumor mass and in the invading areas, **(D2)** BLBP 40x (LC26-RTL(170) 16 weeks post-injection) - extensively expressed in the tumor cells, **(D3, D4)** OTX2 20x, 40x (LC26-RTL(170) 16 weeks post-injection) - extensive expression (over 80% of cells) in tumor cells. TU-tumor; P-parenchyma; PI- perivascular invasion.

As it is imperative from a clinical perspective to investigate BTIC’s fundamental function in brain tumor maintenance, we sought to isolate cells with stem cell characteristics from the tumor mass to get some insight into the biology of cells that are presumably responsible for tumor maintenance. The premise was based on the finding that a NSC specific cell culture condition predominantly facilitates the growth of NSCs as in the case of the RG cells [9], and by using these conditions we should be able to select and expand BTICs from our PNET model tumors. Using this approach, we derived tumor cell lines (TCLs) that morphologically resembled the RG cells (Figure 1B1, 1B2) and demonstrated the expected BTIC features: capacities to self-renew, to differentiate into astrocytes and neurons [10, 11] (Figure 1A1-1A4), and to generate highly invasive tumors once injected in different sites of NOD-SCID mouse brains (Figure 1B3, 1B4, 1C1, 1C2, 1C4, 1D1-1D4, Supplementary Figure 1).

**Figure 2:**
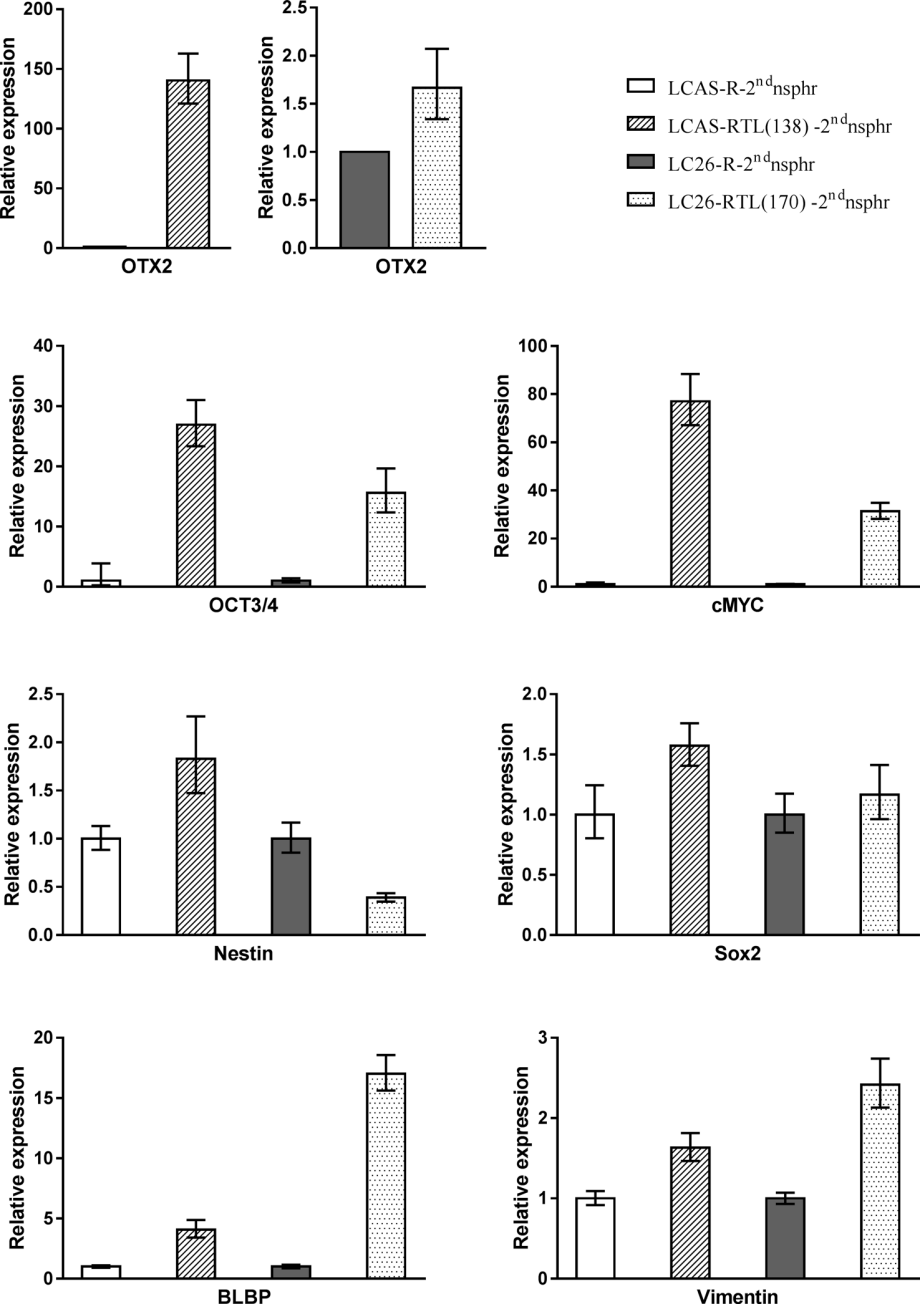
RT-PCR analysis Expression level of OTX2, cMYC, OCT3/4, Nestin, Vimentin, Sox2 and BLBP in the RG and TCL self-renewing cells.

**Figure 3:**
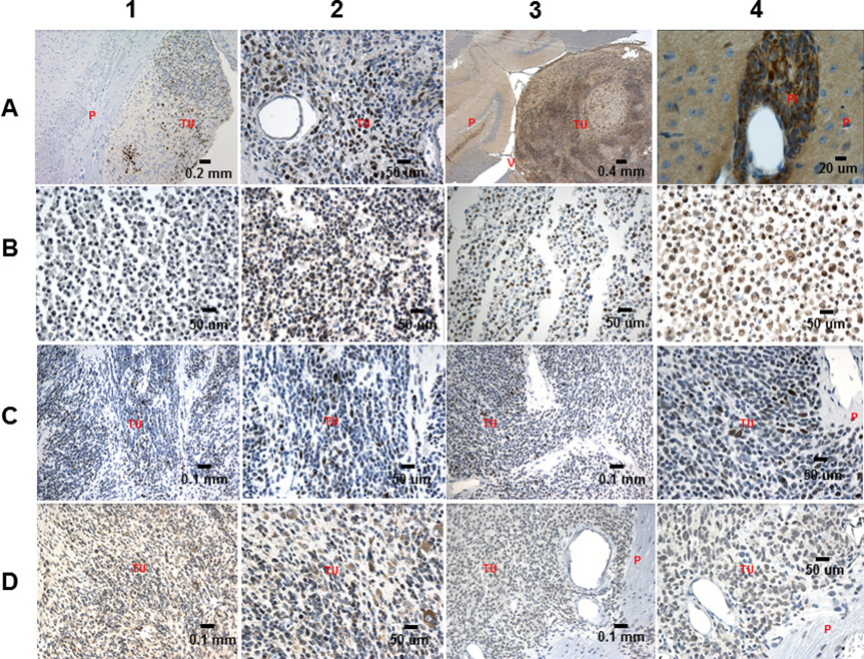
IHC analysis of RG, TCL, the RG and the TCL derived tumors **(A1)** ps62cMYC 10x (LC26-RTL(170) 12 weeks post-injection) - expression is observed in over 25% of tumor cells, **(A2)** cMYC 40x (LCAS-RTL(138) 9 weeks post-injection) - expression is observed in over 25% of tumor cells, **(A3)** EPHA3 5x (LC25-R 8 weeks post-injection) - extensive expression is observed in tumor cells, **(A4)** EPHA3 63x (LCAS-R 12 weeks post-injection) - perivascular invading tumor cells show overexpression of EPHA3, **(B1, B2)** p53 40x (LCAS-R/LCAS-RTL(138)) - B1: show low expression of p53 (<10%) while B2 show over 75% of p53 expressing cells, **(B3, B4)** p53 40x (LC26-R/LC26-RTL(170)) - B3: show low expression of p53 (<20%) while B4 show over 60% of p53 expressing cells, **(C1, C2)** p53 20x, 40x (LCAS-RTL(138) 15 weeks post-injection) - approximately 45% of tumor cells show expression of p53, **(C3, C4)** p53 20x, 40x (LC26-RTL(170) 12 weeks post-injection) - approximately 45% of tumor cells show expression of p53. **(D1, D2)** MDM2 20x, 40x (LCAS-RTL(138) 15 weeks post-injection) - approximately 50% of tumor cells show expression of p53, **(D3, D4)** MDM2 20x, 40x (LC26-RTL(170) 12 weeks post-injection) - approximately 50% of tumor cells show expression of p53. TU-tumor; P-parenchyma; V-ventricle; PI- perivascular invasion.

The TCL’s transcriptome exhibited higher similarity to the RG transcriptome than to the transcriptome of the tumors from which they were derived (Supplementary Figure 2). In comparison to the RG self-renewing cells, the corresponding TCLs showed similar or increased expression of the RG marker BLBP, primitive neuroectodermal marker OTX2, RG-BTIC markers SOX2, Vimentin and Nestin, along with the BTIC marker OCT3/4 (Figure 2). Furthermore, similarly to the tumors generated by the RG cells [8], the TCL generated tumors also demonstrated high expression levels of these markers (Figure 1C1, 1C2, 1C4, 1D1-1D4). Moreover, as was the case for the TCL self-renewing cells, the TCL derived tumors exhibited high expression of onco-protein – cMyc (Figure 2, Figure 3A1, 3A2). Remarkably, the tumors generated by the RG cells [8], the TCLs and the TCL derived tumors exhibited aberrant accumulation of tumor-suppressor p53 (Figure 3B1-3B4, 3C1-3C4). Similarly, as the tumors generated by the RG cells [8], the TCL derived tumors displayed elevated level of p53 inhibitor - MDM2 (Figure 3D1-3D4), which might indicate p53 aberrant modification, rendering resistance to MDM2 mediated degradation [8].

We documented previously that RG cells were unable to generate tumors when injected into the motor cortex or the cerebellum of NOD-SCID mice in contrast to the SVZ of the 3^rd^ ventricle, which resulted in tumors that extensively invaded the brain and the ventricular system [7]. We now report invading tumor cells in different parts of the mouse brain after TCLs injection into the motor cortex or the cerebellum (Supplementary Figure 1).

Altogether, our findings are consistent with the hypothesis that TCLs represent the BTICs in our CNS-PNET model.

### BTIC genomic alterations

Since the least differentiated progenitor cells may require less genetic alterations during the onset of tumorigenesis [12], we hypothesized that in order to become BTICs, the RG cells should acquire minor alterations. Indeed, the analysis of RNA/DNA- seq data from the RG cell lines and the corresponding TCLs revealed that even though some copy number variations (CNV) could be observed in the whole exome sequence data, obtained from the TLCs, as compared to the original RGs, the was no difference in the expression level of the genes residing in the areas affected by CNVs (Supplementary Tables 2-4).

In addition, we found no mutations in the TCLs, except for a few single nucleotide substitutions (Supplementary Tables 5-7). In particular, the comparative sequence analysis of RNA-seq data derived from TP53 in the parental RG and TCLs did not reveal any single nucleotide variants or indels.

### BTIC gene signature

**Figure 4:**
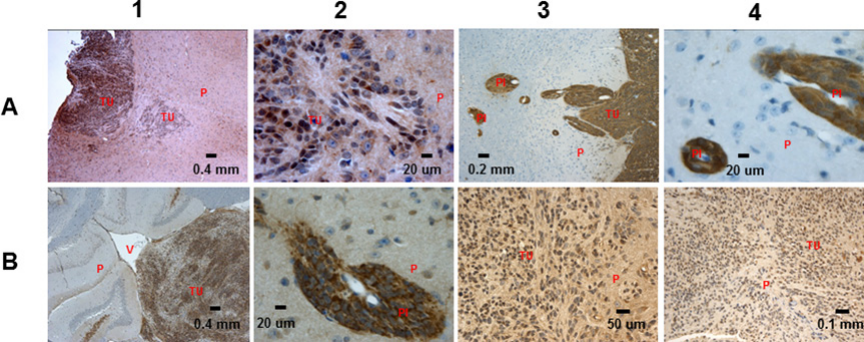
IHC analysis of the RG derived tumors **(A1, A2)** Trim22 5x, 63x (LCAS-R 12 weeks post-injection) - intense expression is observed in all tumor cells, **(A3, A4)** ENO1 10x, 63x (LCAS-R 12 weeks post-injection) - intense expression is observed in all tumor cells, within the main tumor mass and in perivascular invading areas, **(B1, B2)** MALT1 5x, 63x (LC25-R 8 weeks post-injection, LCAS-R 12 weeks post-injection) - intense expression in over 75% of tumor cells within the main tumor mass and in 100% of the perivascular invading cells, **(B3)** Caveolin 1 40x (LCAS-R 12 weeks post-injection) - extensively expressed in tumor cells, **(B4)** Cathepsin C 20x (LC26-R 12 weeks post-injection)- extensively expressed in tumor cells. TU-tumor; P-parenchyma; V-ventricle; PI- perivascular invasion.

Our analysis of the RNA-seq data derived from the CNS-PNET BTICs revealed numerous genes with consistent differential expression relative to the corresponding RG cells and TCLs; it is conceivable that some of these genes play a role in the function of BTICs [13]. Expression of specific genes was verified by IHC on RG generated tumors (Figure 1C3, Figure 3A3, 3A4, Figure 4) and TCL generated tumors (Figure 1C1, 1C2, 1C4, 1D1-1D4, Figure 3A1, 3A2, 3B1-3B4), and/or by RT-PCR (Table 1, Supplementary Table 1) using RG and TCLs second-generation neurospheres [10], which supposedly represent the self-renewing cells of the RG and TCLs. Since genes may be associated with many different functions, we assigned some of the identified genes to more than one of several broad categories: BTIC maintenance, proliferation, migration-invasion, tumor suppressors, anti-apoptosis and metabolism.

**Table 1:** BTIC gene signature verified by RT-PCR on RG and TCLs second-generation neurospheres

Gene	Function	Ref.
**BTIC maintenance**		
BLBP	(RG marker)	[9]
Vimentin (similar)	BTIC maintenance (BTIC marker)	[13]
Sox2 (similar)	BTIC maintenance (BTIC marker)	[10, 14]
Nestin (similar)	BTIC maintenance (BTIC marker)	[10]
OCT3/4	BTIC maintenance (BTIC marker)	[14]
CD44	BTIC maintenance (Glioblastoma multiforme Stem cell marker)	[15]
MYCC	BTIC maintenance	[16]
OTX2	BTIC maintenance	[9, 17]
NR2F1 (down)	Control of tumor cell dormancy	[18]
PRAME	Generation of cancer stem-like cells	[19]
HEY1	NSC maintenance	[20]
Elongin C	Cellular plasticity of pluripotent stem cells	[21]
ANXA1	BTIC maintenance	[22]
EPHA3	BTIC maintenance	[23]
NRP1	BTIC maintenance	[24]
Epigenetic regulators		
KDM4c	BTIC maintenance	[25, 26]
KDM5b	BTIC maintenance	[27-32]
KDM5c	BTIC maintenance	[33]
HDAC9	BTIC maintenance	[34]
**Proliferation**		
MYCC	The key onco-gene, cell proliferation, invasiveness	[35]
MAX	c-Myc co-activator, cell proliferation, invasiveness	[36]
MINA53	c-Myc target gene, cell proliferation	[37]
EMP1	cell proliferation	[38]
EMP3	cell proliferation, motility and invasiveness	[39]
TGFB1	cell proliferation, motility and invasiveness	[40]
ANXA2	regulation of cellular growth	[41]
GTF2H1	transcription elongation from RNA polymerase II promoter	[42]
**Migration-Invasion**		
ENO1	cell invasion, metastatic dissemination	[43]
CTSC	cell invasion	[44, 45]
LOX	cell invasion	[46]
CD44	cell invasion	[47]
MALT1	cell invasion	[48]
TRX1	cell invasion	[49]
**Tumor suppressors**		
TP53 (down)	the key tumor-suppressor	[50]
TRIM22	cell cycle arrest, DNA repair	[51]
DKK3	tumor suppressor gene, antagonizes canonical Wnt signaling	[52]
**Anti-apoptosis**		
PEA15	Anti-apoptosis, regulates glucose transport	[53]
Humanin	Anti-Apoptosis	[54, 55]
**Metabolism**		
CAV1	glycolysis, mitochondrial bioenergetics, fatty acid metabolism	[56]
ACAD11	mitochondrial beta-oxidation of fatty acids	[57]
ACSL3	mitochondrial beta-oxidation of long chain fatty acids	[58]
PPAT	de novo purine biosynthesis	[59]
GART	de novo purine biosynthesis	[60]
ALDOA	a key role in glycolysis and gluconeogenesis	[61]
GLS	a key role in generating energy for metabolism	[62]
TRX1	purine metabolism and glucose/energy metabolism	[49]

((LCAS-R-2^nd^ nsphr/LCAS-RTL(138) -2^nd^ nsphr) and (LC26-R-2^nd^ nsphr/ LC26-RTL(170)-2^nd^ nsphr)).

### BTIC cell energy phenotype

**Figure 5:**
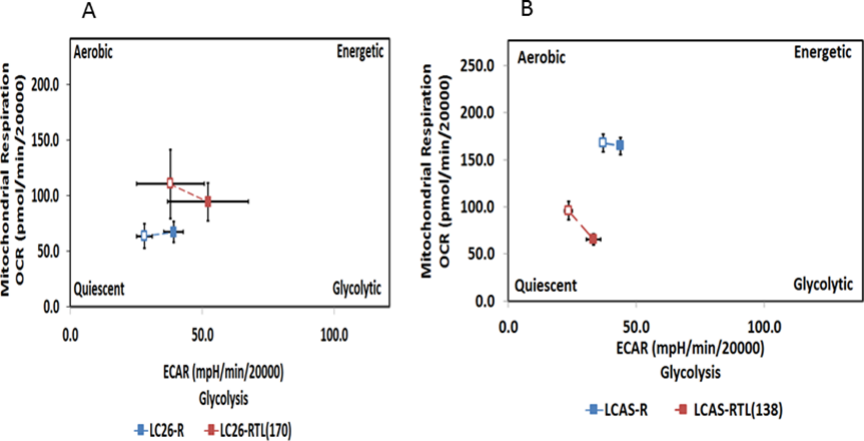
Bioenergetic profiles of the RG and TCL Cell lines LC26-R, LC26-RTL(170), LCAS-R and LCAS-RTL(138) were evaluated for their respective extracellular acidification rate (ECAR) and oxygen consumption rates (OCR) using the phenotype assay kit from Seahorse Bio. **(A)** LC26-R was compared to LC26-RTL, **(B)** LCAS-R was compared to LCAS-RTL.

As the metabolic switch from oxidative phosphorylation to glycolysis, also known as the Warburg effect, has been documented to occur in different types of cancer stem cells [63], we analyzed the oxygen consumption rate (OCR) and extracellular acidification rate (ECAR) [64] in the RGs and TCLs. We found that the TCLs exhibited higher ECAR and lower OCR compared to the correspondent RGs (Figure 5), which indicate that the TCLs derive their energy from glycolysis rather than oxidative phosphorylation.

## DISCUSSION

There is a paradigm of brain tumorigenesis that implicates a limited number of genomic and/or epigenomic alterations in the transformation of neural stem cells (NSC) into brain tumor-initiating cells (BTIC) [2–5]. Yet, the molecular characteristics of BTICs, particularly those of CNS-PNET BTIC, are still largely unknown. As it is imperative from the clinical perspective to investigate PNET BTIC’s function in brain tumor maintenance, we proceeded to isolate cells with NSC characteristics from the tumors originated in our model in order to get insight to the biology of the cells that are presumably responsible for tumor maintenance.

Our results point to the fact that the isolated TCLs may indeed represent the CNS-PNET BTICs, for the following reasons: (1) morphologically the cells resemble the RG cells; (2) they self-renew; (3) they differentiate along the neuronal and glial lineages; and (4) they give rise to tumors once injected in different parts of NOD-SCID mouse brain. The absence of substantial genomic alternations in the TCLs, similar transcriptome profiles of the RGs and TCLs, along with the morphological similarity of the tumors generated by RGs and TCLs suggest that only a limited number of genomic alterations may be required for the tumorigenic transformation of RG cells.

Along with well-established BTIC markers such as Nestin, Vimentin, Sox2 and OCT3/4 [10, 13, 14], we found OTX2 – a medulloblastoma oncogene [17], which we documented previously as an early marker of primitive neuroectoderm and BLBP - an established marker of RG cells, to be highly expressed in the TCL self-renewing cells [9]. Moreover, CD44 - glioblastoma multiforme stem cell marker [15] – is also highly expressed in the TCL self-renewing cells.

We found MYCC onco-gene to be highly expressed in the TCL self-renewing cells, indicating that such overexpression may have taken place in the early stages of the RG tumorigenic transformation along with the overexpression of additional genes (Table 1). Even though abnormal levels of the MYCC onco-gene are detected in the majority of human tumors, the triggering mechanisms for such expression alterations, as well as that of its target genes, are still largely unknown [65], particularly in BTICs. Direct comparison of the RG cells with the TCLs could facilitate decoding of the mechanisms of MYCC altered expression in the RG cells during tumorigenic transformation, in addition to facilitating identification of the corresponding cMYC target genes. Such wide-ranging identification would highlight the pathways underlying cMYC-driven tumorigenesis, thus possibly expediting development of new therapeutic strategies for cMYC-driven tumors. Moreover, our CNS-PNET tumor model could be a powerful tool for screening experimental cMYC - specific drugs, which would facilitate the development of combined therapy regimens [66, 67].

We also identified a plethora of additional transcriptomic changes (Table 1), which could facilitate the BTIC function of tumor maintenance - including self-renewal, cell proliferation, motility and invasiveness, anti-apoptosis or metabolic changes. It is conceivable that some of the up regulated genes could serve as new BTIC markers of CNS-PNET; however, it remains to be determined if these genes are also overexpressed in clinical tumor specimens.

Absence of genomic alterations in the TP53 gene suggests that the accumulation of tumor-suppressor p53 in the TCLs may be due to an aberrant post-transcriptional event instead of CNV or mutation in the gene. In this regard, we found TP53 gene to be significantly down-regulated in the TCL self-renewing cells (Table 1). The absence of genomic alterations in the rest of the genes from the proposed BTIC gene signature (Table 1, Supplementary Table 2-4) indicates also that epigenetic changes may be responsible for the onset and maintenance of CNS- PNET BTICs. Incidentally, we found PRAME, expression of which is normally restricted to testis [19], as being highly expressed in the model tumors and TCL self-renewing cells (Figure 1C3, Table 1). Disruption of the epigenetically controlled tissue specific expression of PRAME may indicate significant perturbations in the epigenetic landscape of RG, potentially taking place during the first steps of the tumorigenic transformation, which could make PRAME a useful BTIC biomarker.

Remarkably, we found epigenetic regulators KDM4c, KDM5b, KDM5c and HDAC9 as being up-regulated in the TCL self-renewing cells. These regulators may play a significant role in the BTIC maintenance [25–34]. It is conceivable that the up- regulation of these epigenetic regulators may be caused by the hypoxic microenvironment that is characteristic of the SVZ of the 3^rd^ ventricle, as we hypothesized in our previous study [8]. Such up-regulation might trigger the majority of the identified transcriptomic changes (Table 1), while enabling tumor formation in the motor cortex and cerebellum (Supplementary Figure 1) - two locations where the injected RG cells did not show any sign of tumorigenic transformation [7]. Further study will be conducted to investigate whether alterations in these epigenetic regulators are also observed in PNET clinical specimens. Such validation may warrant wide-ranging subsequent studies to assess the therapeutic value of epigenetic drugs for the treatment of PNETs [68, 69].

The metabolic switch documented in the TCLs may also play a role in the maintenance of the BTICs supporting the self-renewal and undifferentiated status of the cells [63]. The fact that our model enables direct comparison of RG cells and BTICs makes it invaluable to uncover the molecular mechanisms underlying transformation and the microenvironmental factors contributing to the onset of tumorigenesis and tumor cell invasion. Subsequent studies of the BTICs might also lead to advances in diagnostics and treatment of embryonal brain tumors.

## MATERIALS AND METHODS

### Orthotopic transplantation of RG cells to the Sub-Ventricular Zone (SVZ) of the 3rd ventricle in NOD-SCID mice brain

Transplantations of the LC25-R, LC26-R, and LCAS-R RG cells to the SVZ of 3rd ventricle of the brain of NOD-SCID mice (in average ten mice per each RG cell line) were performed as previously described [7]. Derivation of these RG lines was described previously [7]. Briefly, transplantations of RG cells to target SVZ of 3rd ventricle were performed as follows: a 1.0mm burr hole was made approximately 0.3mm dorsal caudal from the bregma. A 26-gauge needle attached to a 25 μl Hamilton syringe was inserted into the depth of 4.0mm from the skull surface using stereotactic guidance. Five microliters containing ∼200,000 of the RG cells were inoculated into the brain over a period of 10 minutes. The respiratory rate and the anesthetic depth of all animals were monitored every 5 minutes after the surgery by laboratory personnel until the animals had fully recovered from the anesthesia. No adverse events were encountered during the post-operative care. All mice were kept in standard animal husbandry with regular diet in barrier facilities and monitored 2-3 times per week, including recording of their body weight. The mice were sacrificed at 4-16 weeks’ post-inoculation by an i.p. injection of Nembutal Sodium 40-70 mg/kg followed by cervical dislocation, at which time brains were harvested and tumors were resected. The tumor tissues were named LC25-RT, LC26-RT, and LCAS-RT, respectively.

### TCL (Tumor Cell Line) derivation

The tumor tissues LC25-RT, LC26-RT, and LCAS-RT were thoroughly minced, plated and grown in ENStem-A neural expansion medium with FGF2 at 20 ng/ml (Millipore), L-glutamine 2 mM and PenStrep 1× (Gibco) on laminin-coated tissue culture plates at 37°C, 5% CO2 in a humidified atmosphere. Acutase (Millipore) cell detachment was applied before each cell passage. The tumor cell lines were named LC25-RTL(293), LC26-RTL(170), and LCAS-RTL(138), respectively.

### Orthotopic transplantation of TCL cells to brain regions of NOD-SCID mice

Transplantations of the LC26-RTL(170), and LCAS-RTL(138) cells to the SVZ of 3rd ventricle, motor cortex and cerebellum of the brain of NOD-SCID mice (in average ten mice per each TCL) were performed as previously described [7]. Briefly, transplantations of TCL cells to target SVZ of 4th ventricle in cerebellum, motor cortex or SVZ of 3rd ventricle were performed as follows: a 1.0mm burr hole was made approximately -7.0mm dorsal caudal from the bregma, 2.0mm dorsal caudal, 0.8mm right or left lateral from the bregma, and 0.3mm dorsal caudal from the bregma. A 26-gauge needle attached to a 25 μl Hamilton syringe was inserted into the depth of 3.0mm, 2.0mm, and 4.0mm correspondingly from the skull surface using stereotactic guidance. Five microliters containing ∼200,000 of the TCL cells were inoculated into the brain over a period of 10 minutes. The respiratory rate and the anesthetic depth of all animals were monitored every 5 minutes after the surgery by laboratory personnel until the animals had fully recovered from the anesthesia. No adverse events were encountered during the post-operative care. All mice were kept in standard animal husbandry with regular diet in barrier facilities and monitored 2-3 times per week, including recording of their body weight. The mice were sacrificed at 4-16 weeks post-inoculation by an i.p. injection of Nembutal Sodium 40-70 mg/kg followed by cervical dislocation, at which time brains were harvested and perfused with 4% paraformaldehyde as previously described [9].

### Neurosphere culture

The RG and TCL cells were plated at a density of 300 cells per ml on 24 well plates in ENStem-A neural expansion medium with FGF2 at 20 ng/ml (Millipore), L-glutamine 2 mM and PenStrep 1× (Gibco) and grown for 14 days at 37°C, 5% CO2 in a humidified atmosphere. The neurospheres were collected and plated in the same media on laminin-coated tissue culture plates for 24-48 hours (in order to convert the neurospheres into the cell monolayer) at 37°C, 5% CO2 in a humidified atmosphere. Acutase (Millipore) cell detachment was applied and the neurosphere formation process repeated again to produce self-renewal cell culture. The self-renewing cells were named LC26-R-2^nd^ nsphr and LC26-RTL(170)-2^nd^ nsphr, LCAS-R-2^nd^ nsphr and LCAS-RTL(138)-2^nd^ nsphr, respectively.

### Total RNA isolation

Total RNA isolation was performed with the PureZOL RNA isolation reagent (Bio-Rad, Hercules, CA), followed by DNAse treatment (Ambion, Austin, TX), according to the manufacturer’s instructions. Purity and integrity of the isolated RNA was assessed on the ND-1000 Spectrophotometer (Thermo Fisher Scientific, Waltham, MA).

### Total DNA isolation

Total DNA isolation was performed with the Puregene DNA purification kit (Qiagen, Germantown, MD), according to the manufacturer’s instructions. Purity and integrity of the isolated DNA was assessed on the ND-1000 Spectrophotometer (Thermo Fisher Scientific, Waltham, MA).

### RNA-seq and data analysis

RNA-seq library construction was performed according to the manufacturer’s instructions for RiboZero selection. The resulting libraries were sequenced using Illumina HiSeq2500. The FastQC software (http://www.bioinformatics.babraham.ac.uk/projects/fastqc/) was applied on raw fastq files to examine the sequence quality. Tophat [70] was used for tag alignment and counts for each gene were computed by means of HTSeq Python package [71], using the annotation of the Ensembl genes and only reads that mapped to exons. Differential expression analysis on the count data was performed using DESeq2 [72], which is based on a negative binomial distribution and uses shrinkage estimation for the variance of the distribution. As an alternative way of quantifying normalized gene and transcript expression, Fragments Per Kilobase of transcript per Million mapped reads (FPKM) values were also derived using Cufflinks [73] and were furthered normalized by upper quartile normalization (GEO record GSE82102).

Single nucleotide variant (SNV) and small Indel calling were conducted for both RG and TCLs. Alignment files generated by Tophat2 were used for SNV detection using SAMtools [74] and Varscan [75] with the following parameters: map quality>15, PHRED quality score>10, coverage>8 reads, P value threshold for calling variant = 0.01 and minimum supporting reads at a position to call variant = 2. We used ANNOVAR [76] for annotation of the called variants and SAMtools view was used to visualize the aligned reads in the region ofTP53.

RNA-seq PCA: The raw reads count data was normalized by variance stabilizing transformation (VST) method proposed in DESeq2. This method fitted a dispersion-mean relation and then transforms the count data (normalized by division by the size factors or normalization factors. We selected the top 1000 most varied genes using coefficient of variations. The principle component analysis was conducted on the selected genes and ggplot2 package [77] was used to generate the plot.

RNA-seq somatic mutation calling: We applied a method called GLMVC [78] to detect somatic mutations from paired RNA-seq data. This method is based on a bias reduced generalized linear model and showed better performance than MuTect and Varscan on RNA-data.

### Whole exome sequencing data analysis

DNA-seq libraries were sequenced using Illumina HiSeq2500. Paired-end sequencing data from the exome capture libraries were mapped to the reference human genome (build hg19) with BWA aligner [79]. All sequenced and aligned libraries (uniquely mapped reads) were further processed with both the Picard suite (http://sourceforge.net/projects/picard/) and the GATK tools [80], which includes duplicated reads removing, local realignment around indels, base quality recalibration. All these procedures were performed prior to mutation detection. We made use of the EXCAVATOR algorithm [81] to do the CNV detection. EXCAVATOR was run with the default settings. SG-ADVISER [82] was applied to derive functional effects from predicted CNVs.

### Real-time PCR

Total RNA isolation was performed as mentioned above. cDNA synthesis and real-time quantitative reverse transcription-polymerase chain reactions (qRT-PCR) were performed as previously described [8]. QuantStudio 7 instrument (Applied Biosystems, USA) along with PowerUP SYBR Green Master Mix (A25742, Thermo Fisher Scientific, USA) were used according to the manufacturer’s instructions. The PCR conditions were as follows: one cycle at 50°C for 2 min, one cycle at 95°C for 10 min, 40 cycles at 95°C for 15 s, 60°C for 1 min, followed by a melting curve from 60°C to 95°C. Primers were designed using the Primer Express program version 1.5 (Applied Biosystems, CA, USA), and obtained from Integrated DNA Technologies (Coralville, IA, USA) (Supplementary Table 1). 100 nM primers for GUSB RNA (RealTimePrimers.com) were used as an endogenous control for each of the cDNA samples. Comparative Ct method was used to analyze the qRT-PCR (>2X difference in the gene expression level was considered as significant). In the comparative Ct method the QuantStudio 7 software measures amplification of the gene of interest (target) and of GUSB in each cDNA sample. Measurements are normalized using the endogenous control.

### Immunohistochemistry

Formalin-fixed paraffin embedded (FFPE) tumor tissue and RG cell pellets generated in our previous study [8] were used for histological and immunohistochemical analyses. At least two slides (4μm thick) from each FFPE tumor sample were used for the analysis of each antibody presented in this study using standard immunohistochemical methods. The immunohistochemical panel comprised the following antibodies: Anti-Ki-67 (RM-9106, Rabbit monoclonal, 1:200, Thermo scientific), OCT3/4 (H-134) (sc-9081, Rabbit polyclonal, 1:50, Santa Cruz), Anti-Nestin (ab105389, Rabbit monoclonal, 1:30, abcam), Anti-Sox2 (ab97959, Rabbit polyclonal, 1:450, abcam), Anti-Vimentin (NBP1-97671, Mouse monoclonal, 1:500, Novus Biologicals), c-MYC (ab32072, Rabbit monoclonal[Y69], 1:500, abcam), Anti-c-MYC-(Phospho S62) (ab185656, Rabbit monoclonal, 1:500, abcam), p53 (FL-393) (sc-6243, Rabbit polyclonal, 1:200, Santa Cruz), Anti-BLBP (ABN14, Rabbit polyclonal, 1:400, Millipore), Anti-OTX2 (AB9566, Rabbit polyclonal, 1:750, Millipore), Anti-Trim22 (ab140966, Mouse monoclonal, 1:100, abcam), Caveolin (N-20) (sc-894, Rabbit polyclonal, 1:50, Santa Cruz), Cathepsin C/DPPI (AF1034, Goat polyclonal, 1:50, R&D), ENO1 (LS-B10960, Rabbit polyclonal, 1:500, LSBio), Anti-MALT1 (ab93661, Rabbit polyclonal, 1:200, abcam), EPHA3 (LS-C312723, Rabbit polyclonal, 1:200, LSBio), Anti-PRAME (ab135600, Rabbit polyclonal, 1:150, abcam), Anti-MDM2 (LS-C199239, Rabbit polyclonal, 1:100, LS Bio).

### Cell energy phenotype test

The cell energy phenotype assay in the RG and TCL were performed using a Seahorse XFp Cell Energy Phenotype Test Kit (Agilent#103275-100) according to the instruction from the manufacturers. LC26-R, LC26-RTL(170), LCAS-R and LCAS-RTL(138) cells were seeded at 20,000 cells per well in 8-well seahorse cell culture plate and incubated overnight at 37°C in an atmosphere of 5% CO2. Results are represented for two independent experiments. Before analysis, cells were washed twice with sodium bicarbonate- and glucose-free ENStem-A neural expansion medium with FGF2 at 20 ng/ml (Millipore), supplemented with glutamine and penicillin/streptomycin, pH 7.4) and incubated for 1 h at 37°C without CO2. We used FCCP and Oligomycin diluted to 10 μM for determining the cell energy phenotype by recording extracellular acidification rates (ECAR, milli pH/min/20000 cells) and oxygen consumption rates (OCR, pmol/min/20000 cells) on a Seahorse Bioscience Extracellular Flux Analyzer.

## SUPPLEMENTARY MATERIALS FIGURES AND TABLES














