# Supplementary material for: Characterization of brain tumor initiating cells isolated from an animal model of CNS primitive neuroectodermal tumors
Source: Oncotarget. 2018 Feb 9;9(17):13733–47. doi: 10.18632/oncotarget.24460 (PMC5862611; doi:10.18632/oncotarget.24460)
Supplement: Supplementary file 1 [file oncotarget-09-13733-s001.pdf]

Characterization of brain tumor initiating cells isolated from an animal model of CNS primitive neuroectodermal tumors

SUPPLEMENTARY MATERIALS

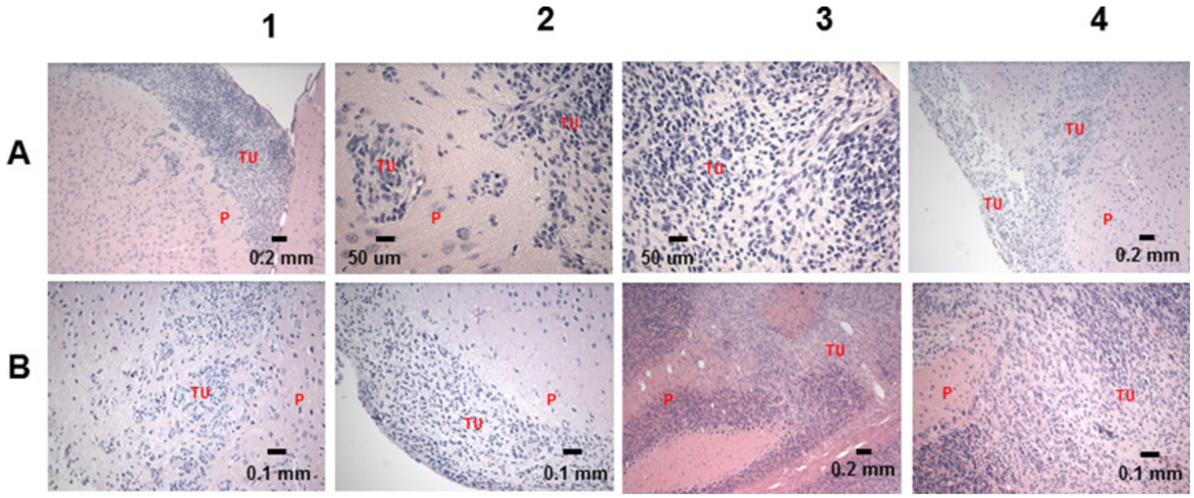

Supplementary Figure 1: Morphological analysis of the TCL derived tumors in motor cortex and cerebellum. (A1) HE 10x (motor cortex: LCAS-RTL(138) 7 weeks post-injection, **(A2)** HE 40x (motor cortex: LCAS-RTL(138) 7 weeks post-injection (invasion area)), **(A3)** HE 40x (motor cortex: LCAS-RTL(138) 7 weeks post-injection (surface area)), **(A4)** HE 10x (motor cortex: LC26-RTL(170) 7 weeks post-injection - superficial growth of a tumor characterized by undifferentiated small round blue cells. Foci of perivascular invasion of tumor cells is observed within the adjacent brain parenchyma; **(B1)** HE 20x (motor cortex: LC26-RTL(170) 7 weeks post-injection (invasion area)), **(B2)** HE 20x (motor cortex: LC26-RTL(170) 7 weeks post-injection (surface area)), **(B3)** HE 10x (cerebellum: LC26-RTL(170) 7 weeks post-injection, **(B4)** HE 20x (cerebellum: LC26-RTL(170) 7 weeks post-injection - a tumor mass grows superficially in motor cortex, extensive invades the adjacent, brain parenchyma and disseminates throughout the ventricular system reaching the cerebellum and extensively infiltrating it.

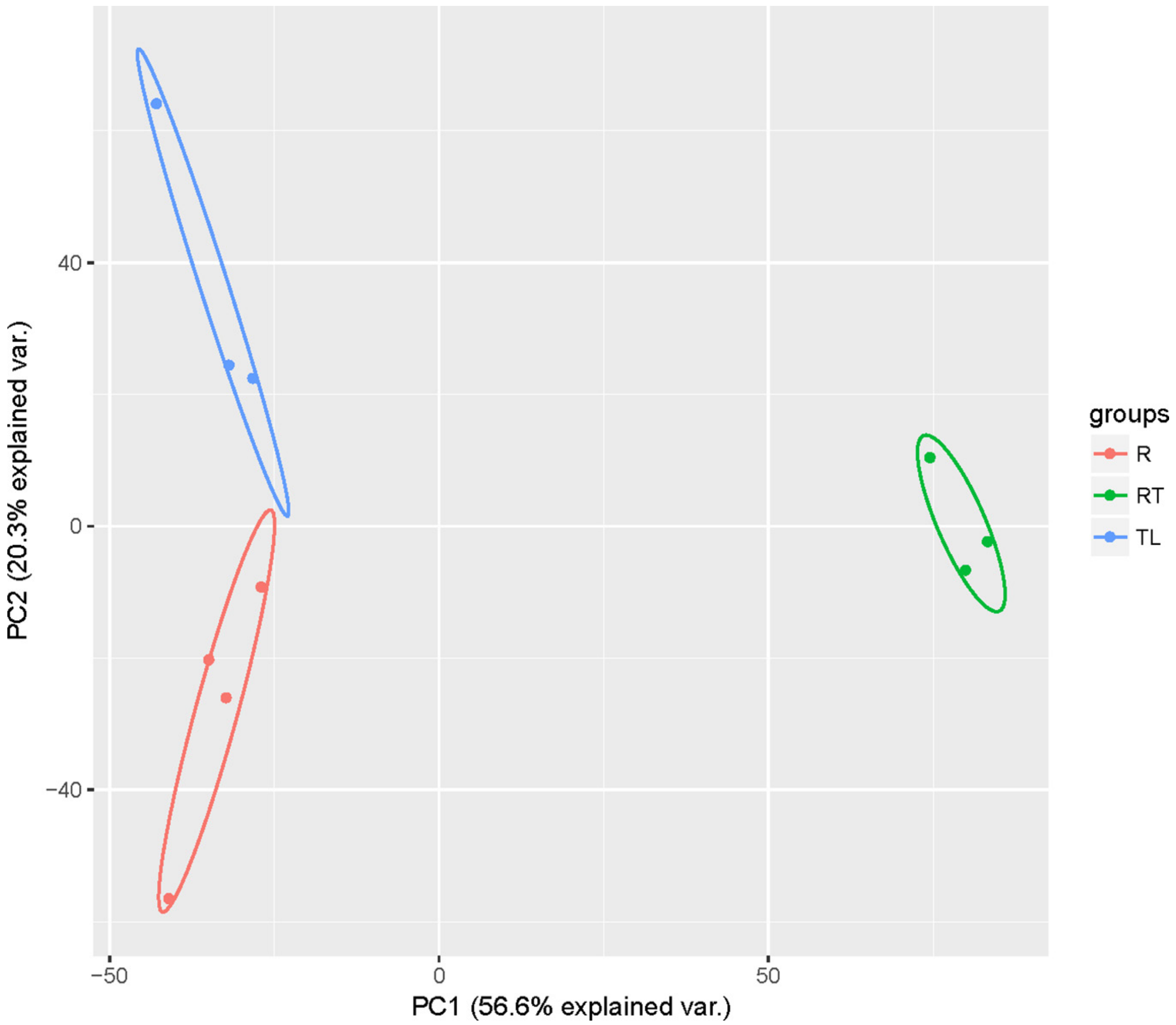

Supplementary Figure 2: RNA-seq PCA. Score plot of the first principal component (PC1) versus second principal component PC2 from PCA of the 1000 most varied genes. These two components describe around 77% of the total variation. PC1 explains 56.6% of the total variation and demonstrates a grouping of TL and R samples, separated with RT. PC2 distinguishes the three TL samples with the three R samples.

Supplementary Table 1: RT-PCR primer sequences

Gene	Forward primer (5’ → 3’)	Reverse primer (5’ → 3’)
CD44	CTGCCGCTTTGCAGGTGTA	CATTGTGGGCAAGGTGCTATT
cMYC	CCTACCCTCTCAACGACAGC	TTGTTCCTCCTCAGAGTCGC
Vimentin	AGTCCACTGAGTACCGGAGAC	CATTTCACGCATCTGGCGTTC
Sox2	GCCGAGTGGAAACTTTTGTCG	GGCAGCGTGTACTTATCCTTCT
BLBP	GTGGGAAATGTGACCAAACC	CTTTGCCATCCCATTTCTGT
Nestin	CAGGAGAAACAGGGCCTACA	TGGGAGCAAAGATCCAAGAC
OCT3/4	GGAAGGTATTCAGCCAAACG	CTCCAGGTTGCCTCTCACTC
OTX2	CATGCAGAGGTCCTATCCCAT	AAGCTGGGGACTGATTGAGAT
NR2F1	AAAGCCATCGTGCTGTTCAC	TGCAGGCTCTCGATGTGG
Prame	CAGGACTTCTGGACTGTATGGT	CTACGAGCACCTCTACTGGAA
HEY1	GTTCGGCTCTAGGTTCCATGT	CGTCGGCGCTTCTCAATTATTC
Elongin C	CATCAGGCACGATAAAAGCCA	GCTGTTAGTGTAGCGAACCTTG
ANXA1	CTAAGCGAAACAATGCACAGC	CCTCCTCAAGGTGACCTGTAA
ANXA2	CTCTACACCCCCAAGTGCAT	TCAGTGCTGATGCAAGTTCC
Humanin	TGACACATGTTTAACGGCCG	AGGGRCTTCTCGTCTTGCTG
EPHA3	ACTCTACGAGACTGCAATAGCA	TCCCCAAGARCCATTTGAGTG
NRP1	ACGTGGAAGTCTTCGATGGAG	CACCATGTGTTTCGTAGTCAGA
KDM4c	CGAGGTGGAAAGTCCTCTGAA	GGGCTCCTTTAGACTCCATGTAT
KDM5b	AGTGGGCTCACATATCAGAGG	CAAACACCTTAGGCTGTCTCC
KDM5c	TCAGTGACAGTAAACGGCACC	ACACCGGCATCACATTTAGGT
HDAC	AGTAGAGAGGCATCGCAGAGA	GGAGTGTCTTTCGTTGCTGAT
MAX	GGAGAGCGACGCTGACAAAC	AGGTGTGGCATTTCTGCATC
MINA53	CCACATACTATGGGTCCCTGT	TGAAACTGAATCGTTGCCCTT
EMP1	CCACTTCTGGGTCATGCACT	TTGCCAGAACTTGTACCGCT
EMP3	CCTGAATCTCTGGTACGACTGC	GCCATTCTCGCTGACATTACTG
TGFB1	CTTCGCCCCTAGCAACGAG	TGAGGGTCATGCCGTGTTTC
ANXA2	CTCTACACCCCCAAGTGCAT	TCAGTGCTGATGCAAGTTCC
GTF2H1	GACCTTGTTGTGAGTCAAGTGA	CCTGCTTATGATTGGATGTGGAA
ENO1	TGATCGAGATGGATGGAACA	GCAGGATGACTTCAGAGTTGC
CTSC	TTGTTCATGCAGCTGGGAAC	TCACCAGGACTCCTTTGCAT
LOX	CGGCGGAGGAAAACTGTCT	TCGGCTGGGTAAGAAATCTGA
MALT1	ACCGCCAATATTGTGTTTGG	TGCAACTTCATCCAGTAACACA
TRX1	GTGAAGCAGATCGAGAGCAAG	CGTGGCTGAGAAGTCAACTACTA
P53	GCTTCCCTGGATTGGCAGC	GAGCTTCATCTGGACCTGGG
TRIM22	CTGTCCTGTGRGRCAGACCAG	TGTGGGCTCATCTTGACCTCT
DKK3	AGGACACGCAGCACAAATTG	CCAGTCTGGTTGTTGGTTATCTT
PEA15	GGAGAGCCACAACAAGCTG	CCATAGTGAGTAGGTCAGGACG
CAV1	ACCCACTCTTTGAAGCTGTTG	GAACTTGAAATTGGCACCAGG
ACAD11	TTGGATTCCCCGTTCCCAAG	AAATCACGGAAGATTCGACCC
ACSL3	ATGGAAAACCAACCTCATAGCAA	GCCATCCCAGTTATACCAGCAA
PPAT	AATTGTCAGCCCTTCGTTGTT	CCTTAATCGAGCAGCATTTACCA
GART	GTGTCTGTGTTTCACTGATGGC	GCTGGACAATAGGCTCCCATT
ALDOA	CAAGAAGGACGGAGCTGACT	TTCAAGTCATGGTCCCCATC
GLS	TCTACAGGATTGCGAACGTCT	CTTTGTCTAGCATGACACCATCT

Supplementary Table 2: Copy number variations (CNV) and the expression level of the genes resided in the areas affected by CNVs (LC26-RTL(170)

See Supplementary File 1

Supplementary Table 3: Copy number variations (CNV) and the expression level of the genes resided in the areas affected by CNVs (LC25-RTL(293)

See Supplementary File 2

Supplementary Table 4: Copy number variations (CNV) and the expression level of the genes resided in the areas affected by CNVs (LCAS-RTL(138)

See Supplementary File 3

Supplementary Table 5: Single nucleotide polymorphism (SNP): LC25-RTL(293)

See Supplementary File 4

Supplementary Table 6: Single nucleotide polymorphism (SNP): LC26-RTL(170)

See Supplementary File 5

Supplementary Table 7: Single nucleotide polymorphism (SNP): LCAS-RTL(138)

See Supplementary File 6
